# Childhood craniopharyngioma: a retrospective study of children followed in Hôpital Universitaire de Bruxelles

**DOI:** 10.3389/fendo.2024.1297132

**Published:** 2024-06-19

**Authors:** Clémentine Magerman, Emese Boros, Marco Preziosi, Sophie Lhoir, Nathalie Gilis, Olivier De Witte, Claudine Heinrichs, Isabelle Salmon, Christophe Fricx, Françoise Vermeulen, Laetitia Lebrun, Cécile Brachet, Marine Rodesch

**Affiliations:** ^1^ Université Libre de Bruxelles (ULB), Hôpital Universitaire de Bruxelles (HUB), CUB Hôpital Erasme, Department of Pediatrics, Brussels, Belgium; ^2^ Université Libre de Bruxelles (ULB), Hôpital Universitaire de Bruxelles (HUB), Hôpital Universitaire des Enfants Reine Fabiola (HUDERF), Pediatric Endocrinology Unit, Brussels, Belgium; ^3^ Université Libre de Bruxelles (ULB), Hôpital Universitaire de Bruxelles (HUB), Hôpital Universitaire des Enfants Reine Fabiola (HUDERF), Pediatric imaging Department, Brussels, Belgium; ^4^ Université Libre de Bruxelles (ULB), Hôpital Universitaire de Bruxelles (HUB), Hôpital Universitaire des Enfants Reine Fabiola (HUDERF), Department of Ophthalmology, Brussels, Belgium; ^5^ Université Libre de Bruxelles (ULB), Hôpital Universitaire de Bruxelles (HUB), CUB Hôpital Erasme, Department of Neurosurgery, Brussels, Belgium; ^6^ Université Libre de Bruxelles (ULB), Hôpital Universitaire de Bruxelles (HUB), CUB Hôpital Erasme, Department of Pathology, Brussels, Belgium; ^7^ DIAPath, Center for Microscopy and Molecular Imaging (CMMI), ULB, Gosselies, Belgium

**Keywords:** childhood craniopharyngioma, neurosurgery, radiotherapy, hypothalamic obesity, endocrine disorders, visual disorders

## Abstract

**Introduction:**

Craniopharyngiomas (CPs) are benign brain tumors accounting for 5 - 11% of intracranial tumors in children. These tumors often recur and can cause severe morbidity. Postoperative radiotherapy efficiently controls and prevents progression and recurrence. Despite advancements in neurosurgery, endocrinological, visual, and neuropsychological complications are common and significantly lower the quality of life of patients.

**Methods:**

We performed a retrospective study, including all patients younger than sixteen diagnosed with CP between July 1989 and August 2022 and followed up in Hôpital Universitaire de Bruxelles.

**Results:**

Nineteen children with CP were included, with median age of 7 years at first symptoms and 7.5 at diagnosis. Common symptoms at diagnosis were increased intracranial pressure (63%), visual impairment (47%), growth failure (26%), polyuria/polydipsia (16%), and weight gain (10.5%). As clinical signs at diagnosis, growth failure was observed in 11/18 patients, starting with a median lag of 1 year and 4 months before diagnosis. On ophthalmological examination, 27% of patients had papillary edema and 79% had visual impairment. When visual disturbances were found, the average preoperative volume was higher (p=0.039). Only 6/19 patients had gross total surgical resection. After the first neurosurgery, 83% experienced tumor recurrence or progression at a median time of 22 months. Eleven patients (73%) underwent postsurgical radiotherapy. At diagnosis, growth hormone deficiency (GHD) was the most frequent endocrine deficit (8/17) and one year post surgery, AVP deficiency was the most frequent deficit (14/17). Obesity was present in 13% of patients at diagnosis, and in 40% six months after surgery. There was no significant change in body mass index over time (p=0.273) after the first six months post-surgery.

**Conclusion:**

CP is a challenging brain tumor that requires multimodal therapy and lifelong multidisciplinary follow-up including hormonal substitution therapy. Early recognition of symptoms is crucial for prompt surgical management. The management of long-term sequelae and morbidity are crucial parts of the clinical path of the patients. The results of this study highlight the fundamental importance of carrying out a complete assessment (ophthalmological, endocrinological, neurocognitive) at the time of diagnosis and during follow-up so that patients can benefit from the best possible care.

## 1 Introduction

Craniopharyngiomas (CPs) are benign slow growing brain tumors (WHO grade I) originateing from remnants of the Rathke’s pouch. CPs are typically located in the sellar and suprasellar regions. The annual incidence of CP ranges from 0.5 to 2.5 new cases per million individuals. The majority (30 - 50%) of cases are diagnosed in children younger than eighteen (1, 2). There is no significant difference in occurrence between sexes (1–4).

There are two histological subtypes of CP, with different tumoral pathogenesis and age distributions: adamantinomatous CP (ACP), associated with somatic variants in the *CTNNB1* gene, with a bimodal peak of incidence, occurring in the 5-15-year and 45-60-year age groups; papillary CP (PCP), associated with somatic variants in the *BRAF V600E* gene (1, 2, 5, 6) most often occurring in the fifth and sixth decades of life (1). *CTNNB1* gene encodes for β-catenin which is involved in cellular pathways of proliferation and survival. *BRAF V600E* gene variants can lead to the activation of the mitogen-activated protein kinase (MAPK) signaling pathway and contribute to dysregulated cell proliferation (1, 2, 5, 6).

The clinical presentation of CP is insidious and heterogeneous, and is related to its location near the hypothalamus, third ventricle, pituitary gland, and optic chiasm. The most frequent manifestations include signs of increased intracranial pressure due to hydrocephalus, visual impairment, and endocrine-related signs (growth retardation, polyuria, polydipsia) (1, 2). The diagnosis of CP in childhood if typically delayed by a few years (1, 4, 5).

From all this, it results that CP, although histologically benign, is a clinically aggressive tumor, associated with high morbidity and occasional mortality.

Surgical management remains a controversial topic, but surgery is still the first treatment option in pediatric craniopharyngiomas. Many studies showed that gross total resection was the most important risk factor for hypothalamic dysfunction (7). Nowadays the objective of surgery is to achieve the most extensive surgical resection which permits to preserve the integrity of hypothalamic and crucial neurological structures (such as the optic nerve, chiasm, third ventricle). The degree of surgical resection depends on preoperative hypothalamic involvement according to Müller’s classification (1, 2). Müller’s classification is a valuable tool for assessing CP, specifically regarding the extent of hypothalamic invasion by the tumor.

By employing this radiological grading system, surgeons can tailor the surgical approach (transcranial, transsphenoidal) and aim at the preservation of hypothalamic functions while striving for optimal tumor removal to minimize the risk of recurrence. Hypothalamic sparing surgery is recommended if there is hypothalamic involvement (grade 1 or 2), while gross total resection is recommended for patients with grade 0 tumors (1, 2, 4, 5). Radiotherapy (photon or proton therapy) for residual tumor control is an important adjunct. Despite recent advances in management aiming to limit morbidity, CP is associated with high morbidity. Data from pediatric series show a 5-year survival rate of 83 to 96%, decreasing to 65 to 100% after 10 years and stabilizing at 62% on average after 20 years (2).

Morbidity after treatment, is mainly determined by the degree of hypothalamic dysfunction and visual disturbances (8, 9). Hormonal deficiencies, obesity and cognitive disturbances are the main hypothalamic dysfunctions. Common manifestations of visual pathway damage include reduced visual acuity and visual field defects, typically presenting as bitemporal hemianopia, strabismus and/or abnormal pupillary responses (papilledema, optic nerve atrophy at the fundus). Hormonal deficits are treated by restoring hormone, but hypothalamic obesity and behavioral changes are difficult to manage, especially in the presence of visual disturbances. In a recent study on 87 patients with CP, 90.8% had one or more pituitary deficiencies at follow-up (10). In the same study BMI outcomes seemed to be better in the last years, after centralization of care, but no differences in visual disturbances and hormonal deficiencies were noted over time.

Along with surgical resection, lifelong hormonal substitution, and management of long-term sequalae and morbidity are important parts of treatment. Despite technical advances in neurosurgery, endocrinological (hypopituitarism, hypothalamic obesity, AVP-D), visual, and neuropsychological complications are common and significantly worsen the quality of life of these patients (1, 11). Lifelong multidisciplinary follow-up is necessary, and clinical trials involving targeted therapies show promise for reducing the risk of these complications (6, 12).

## 2 Materials and methods

This retrospective and descriptive study was conducted at Hôpital Universitaire de Bruxelles (HUB). We included nineteen patients who were diagnosed with CP before the age of sixteen who were followed by a multidisciplinary team (pathologist, pediatrician, endocrinologist, ophthalmologist, neurosurgeon, and neuro-oncologist) at HUB. The study period spanned from July 1989 to August 2022, a 33-year period. The HUB being a tertiary reference center for the management of intracranial tumors. Three Algerian patients initially treated in their country were referred to HUB for further treatment.

This study was approved by the Ethics Committee of HUB (P2023/155).

**Figure 1:**
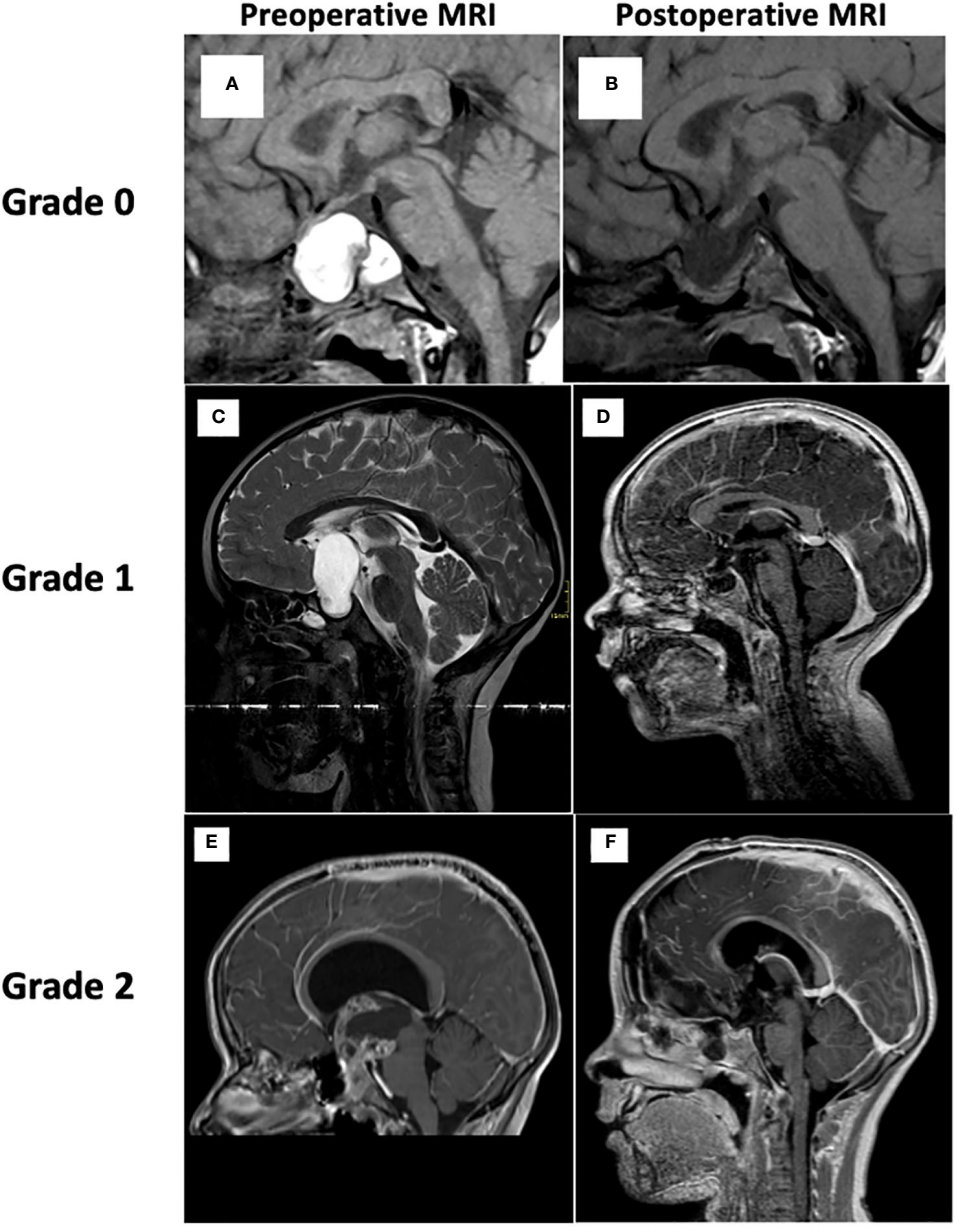
Brain MRI and grading according to Müller’s classification. Grade 0. Reference from: Otte A, Müller HL. Childhood-onset Craniopharyngioma. J Clin Endocrinol Metab. 27 sept 2021;106(10):e3820−36. **(A)** Preoperative sagittal T1-WI shows a predominantly cystic lesion in the sella turcica and the suprasellar cistern. No mass effect is visible on the infundibulum, the floor of the third ventricle or the mamillary bodies. **(B)** Postoperative sagittal T1-WI. Grade 1. **(C)** Preoperative sagittal T2-WI shows a predominantly cystic mass of the hypothalamic-pituitary region pushing back the floor of the third ventricle (no mass effect on the mamillary bodies). **(D)** Postoperative sagittal T1-WI with Gadolinium shows a complete resection of the tumor (no tumor residue). Grade 2. **(E)** Preoperative sagittal T1-WI after contrast administration, shows a solid and cystic mass in the hypothalamo-pituitary region compressing and invading the anterior and posterior hypothalamus. **(F)** Postoperative sagittal T1-WI after gadolinium, shows mass effect resolution. *MRI, magnetic resonance imaging; WI, weighted imaging*.

Demographic and anthropometric data, clinical presentation at diagnosis, ophthalmological impairment, and endocrine disorders pre- and postoperatively (at 6 months, and 1 year) were analyzed. Visual disorders (unilateral or bilateral decrease in visual acuity, color vision and/or visual field deficit, bitemporal hemianopsia, homonymous lateral hemianopsia) were assessed through measurements of visual acuity, visual field, optical coherence tomography, and ocular fundus to find any papilledema or optic nerve atrophy. Endocrine assessments included anamnestic data (polyuria, polydipsia), growth curve analysis (13), pubertal assessment, basal and stimulated hormonal status, and hormonal substitution therapy. Central hypothyroidism was diagnosed if thyroid-stimulating hormone (TSH) was low-normal and/or free thyroxine (FT4) was low. Central adrenal insufficiency was determined by low fasting cortisol or low peak cortisol during glucagon test. Central hypogonadism was defined as the need for hormonal substitution to induce puberty. Growth failure was defined by a decrease in height velocity compared to the reference curves. In the immediate post-operative period, somatotropic axis was assessed through IGF1 dosage and a glucagon test was performed during the first-year post-surgery. Growth hormone secretion was tested by a glucagon stimulation test in cases of growth failure, using 0.1 mg/kg with a maximum of 2 mg of intramuscular glucagon as stimulus. The growth hormone cut-off was 7 mcg/l after 2011 and 10 mcg/l before 2011. The glucagon test results were interpreted taking into account the BMI SDS score, height velocity and IGF1 plasma level (14). In prepubertal children older than 9 years in girls and 10 years in boys, a sex steroid priming was administered (1mg/kg Testosterone esters im 5 days before the test in boys and 1 mg 17 beta estradiol orally for three consecutive days before the test in girls). For IGF1, age specific cut-offs were used. The assay used for growth hormone and IGF1 changed over time: after 2011 they were measured by a sandwich chemiluminescence immunoassay (LIAISON^®^ XL). Body mass index (BMI) and height were expressed as standard deviation score (SDS) according to Cole et al., 1995 (13). Obesity was defined as a BMI above 2 SDS. A single neuroradiologist conducted a retrospective evaluation of preoperative and postoperative brain MRI or CT to determine the tumor volume and grade according to Müller’s classification. This classification includes three distinct grades: Grade 0 is defined by exclusive intrasellar and suprasellar involvement of the tumor (mainly in the chiasmatic cistern without extension to the hypothalamic structures), and the tumor is located entirely outside the hypothalamus. Grade 1 is given to CP with anterior hypothalamic involvement or compression. Grade 2 is given to CP involving the whole hypothalamus (including mamillary bodies), as assessed by brain MRI (1, 2) (**Figure 1**). The tumor site (intrasellar and/or suprasellar), the primary surgical approach (transcranial or transsphenoidal), the histological subtype of CP (adamantinomatous or papillary) and the first therapeutic intervention were recorded. Gross total resection (GTR) was defined as complete macroscopic removal of the tumor assessed by MRI, subtotal resection (STR) as tumor residue less than 10%, and partial resection as a tumor residue of more than 10%. CP recurrence or progression was defined as postoperative growth of the tumor residue observed on MRI. Radiotherapy (proton therapy or gamma knife) was administered to children with tumor progression or recurrence after multidisciplinary discussion. Proton therapy is used since 2011. The time lag between the first surgery and recurrence or progression was documented. Pathological diagnosis and molecular testing were performed in the Department of Pathology of the HUB. The molecular testing was performed using Next Generation Sequencing (NGS) technology with a 50 genes cancer panel including among other *CTNNB1* and *BRAF* genes. Sufficient formalin-fixed paraffin-embedded (FFPE) material was necessary to perform the molecular testing.

### 2.1 Statistical methods

Continuous variables are summarized by the median with range or mean ± standard error of the mean (SEM) and qualitative variables as n (%). ANOVA for repeated measures using the Huynh-Feldt test followed by the least significant difference *post hoc* comparison was used to assess the evolution of variables over time. Linear regression was used to investigate the association between 2 continuous variables. Associations between 2 categorical variables were tested with Fisher’s exact test using the chi-square statistic. Statistical significance was accepted when *p* was <0.05. All statistical tests were two-sided and performed using IBM-SPSS (version 28.0) software (IBM Corp, Armonk, NY, USA).

## 3 Results

The baseline characteristics of the patients and tumors are shown in **Table 1**.

**Table 1:** Baseline characteristics of the patients and tumors.

	All patients n = 19
**Sex ratio**	1.7
Female n (%)	7 (37)
Male n (%)	12 (63)
**Median age at first symptoms in years [range]**	7 [1.6 – 13]
**Median age at diagnosis in years [range]**	7.6 [2.10 – 13.5]
Clinical symptoms at diagnosis n (%)	
Headache	12 (63)
Vomiting	12 (63)
Polyuria/polydipsia	3 (16)
Growth failure	5 (26)
Visual impairment	9 (47)
Weight gain	2 (10.5)
Tumor site n	
Intrasellar	2
Suprasellar	7
Intra + Suprasellar	10
Tumor characteristics n	
Cystic and solid	17
Only cystic	2
Calcifications	17
Müller’s classification n	
Grade 1	3/13
Grade 2	10/13
Tumor volume	
Preoperative, mean +- SEM in *cm3* [range]	17.54 +- 3.65 [2.61 – 39.53]
Postoperative, mean +- SEM in *cm3* [range]	6.00 +- 2.18 [0 – 20.95]
Hydrocephalus at diagnosis n	6
Ventriculo-peritoneal shunt n	6
Primary surgical approach n	
Transsphenoidal	7/16
Transcranial	9/16
First therapeutic intervention n	
Gross total resection	6
Subtotal resection	8
Partial resection	3
Interferon-α	1
Radioactive yttrium-90	1
**Marsupialization of the cyst** n	3/19
Histology n	
Adamantinomatous	11/11
Missing data	8
Molecular biology n	
*CTNNB1* mutation	4/5
No mutation detected	1/5
Recurrence or progression after first surgery n	15/18
Time lag between first surgery and recurrence or progression in months, median [range]	22 [2 - 101]
If recurrence or progression, second therapeutic intervention n	
Surgery alone	3/15
Surgery + RT	7/15
RT alone	4/15
MRI follow-up	1/15
Type of radiotherapy n	
Proton therapy	8
Gamma knife	3
The follow-up period in months, median [range]	157.5 [1 - 397]

SEM, standard error of the mean; RT, radiotherapy; MRI, magnetic resonance imaging.

### 3.1 Patient characteristics

Nineteen children (7 females and 12 males), including 3 patients referred from Algeria, with histologically proven CP were included. The median age at first symptoms was 7 years (range [1 year 6 months – 13 years]), and the median age at diagnosis was 7 years 6 months (range [2 years 10 months – 13 years 5 months]). The clinical symptoms at diagnosis were principally increased intracranial pressure (headache and vomiting) (63%), visual impairment (47%), growth failure (26%), polyuria and polydipsia (16%), and weight gain (10.5%). One patient was diagnosed with a CP during an evaluation for congenital Claude-Bernard Horner syndrome an association reported only once in the literature (15). The median follow-up period was 157.5 months (range [1 - 397]).

### 3.2 Tumor characteristics

An exclusively intrasellar location was noted in two patients, whereas ten had both intrasellar and suprasellar tumors. Six children presented with hydrocephalus at diagnosis. Three patients had a Müller grade 1 tumor, and ten had a grade 2 tumor. There were six patients for whom we lacked grade information (no preoperative MRI available in our possession). Seventeen patients had both a cystic and a solid CP, with calcifications in seventeen patients. Adamantinomatous CP was confirmed histologically for the eleven patients with available pathological results. A *CTNNB1* mutation was identified in 4 cases by next-generation sequencing (NGS). One patient had no detected mutation, and the remaining 14 patients did not have enough material to detect a mutation or were managed before NGS became routine in clinical practice.

### 3.3 Treatment characteristics

Out of 16 patients, the transcranial approach was performed in 9 patients, and transsphenoidal approach was performed in 7 patients as the primary surgical approach. GTR was carried out in 6 patients, STR in 8 and partial resection in 3 patients. All patients with hydrocephalus at diagnosis underwent a ventriculoperitoneal shunt. Among the ten patients with grade 2 tumors, 6 had an STR, 1 had a GTR in Algeria, 1 had a partial resection, 1 received intracystic radioactive yttrium-90 injection in Algeria, and 1 underwent intracystic interferon-α treatment in Algeria. For the 3 patients with grade 1 tumors, GTR, STR and partial resection were performed.

Recurrence or progression of the tumor occurred in 16/18 patients after the first surgery, within a median time of 22 months (range [2-101 months]). As a second therapeutic intervention (in 15 patients), 7 patients had a second surgery followed by postoperative radiotherapy, 4 only postoperative radiotherapy and 2 only a second surgery. Out of the 11 patients who had radiotherapy, 8 received proton therapy and 3 gamma knife. None received photon therapy.

### 3.4 Endocrine deficits and hormonal substitution

**Figure 2:**
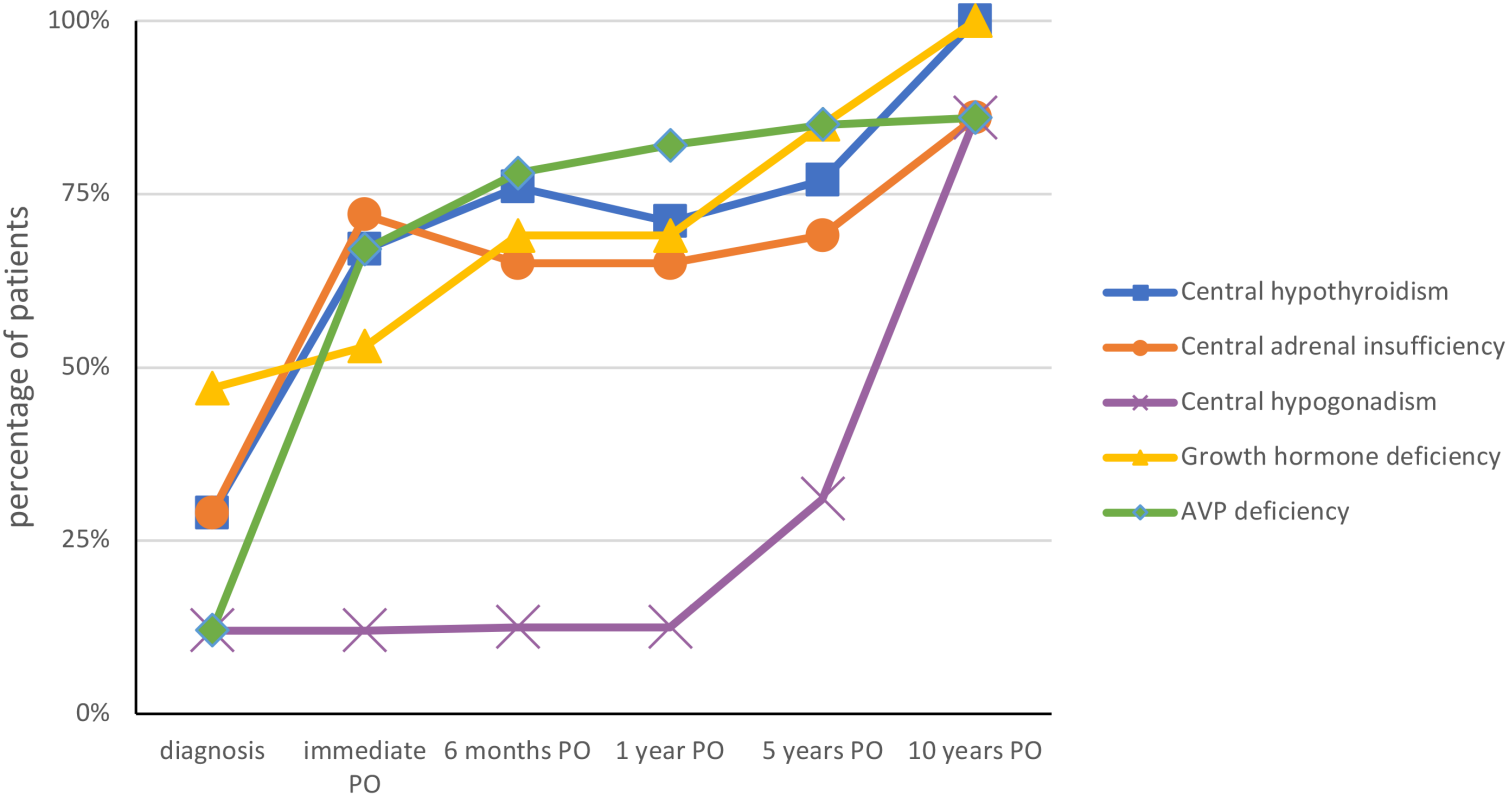
Evolution of endocrinological deficits at diagnosis and after surgery. *PO, postoperative*.

Hormonal deficiencies at diagnosis and at the immediate, 6-month, 1-year, 5 years and 10 years postoperative assessments are summarized in **Table 2**; **Figure 2**.

**Table 2:** Endocrine deficits at diagnosis and after surgery.

Affected pituitary axes	At diagnosis n (%)	Immediate postoperative n (%)	After 6 months n (%)	After 1 year n (%)	After 5 years n (%)	After 10 years n (%)
Central hypothyroidism	5/17 (29)	12/18 (67)	13/17 (76)	12/17 (71)	10/13 (77)	7/7 (100)
Central adrenal insufficiency	5/17 (29)	13/18 (72)	11/17 (65)	11/17 (65)	9/13 (69)	6/7 (86)
Central hypogonadism	2/17(12)	2/17 (12)	2/16 (12,5)	2/16 (12,5)	4/13 (31)	6/7 (86)
Growth hormone deficiency	8/17 (47)	9/17 (53)	11/16 (69)	11/16 (69)	11/13 (85)	7/7 (100)
AVP deficiency	2/17 (12)	12/18 (67)	14/18 (78)	14/17 (82)	11/13 (85)	6/7 (86)

The total number of patients is specified due to missing data or loss of follow-up.

AVP-D, Arginine vasopressine deficiency.

At diagnosis, eleven of 18 patients (61%) had growth failure. The median age at the start of growth failure was 5 years 5 months (range [1 year 2 months – 13 years 3 months]). The median time lag between growth failure and diagnosis of CP was 1 year 4 months (range [0 months – 5 years 6 months]).

At diagnosis, 9/17 patients had at least one hormonal deficiency (thyreotrope, corticotrope, somatotrope, gonadotrope). Eight of 17 patients (47%) had hypo- or hypernatremia in the immediate postoperative period. Two patients had immediate post-operative central adrenal insufficiency which was the result of exogenous steroid administration and did not persist. At one year postoperatively, 15/17 patients (88%) exhibited at least one hormonal deficiency. All patients with central hypothyroidism, central adrenal deficiency, and AVP-D were treated with L-thyroxine (L-T4), hydrocortisone and desmopressin (DDAVP), respectively. Among the 11 patients with growth hormone deficiency, 7 were treated with recombinant growth hormone (rGH) 1 year after surgery, whereas in 4 patients, radiotherapy, migration or tumor progression retarded GH treatment. GH was stopped in case of relapse and restarted 6-12 months after completion of relapse treatment.

There was no association between postoperative endocrine disorders and the type of surgery.

### 3.5 Hypothalamic dysfunction

**Figure 3:**
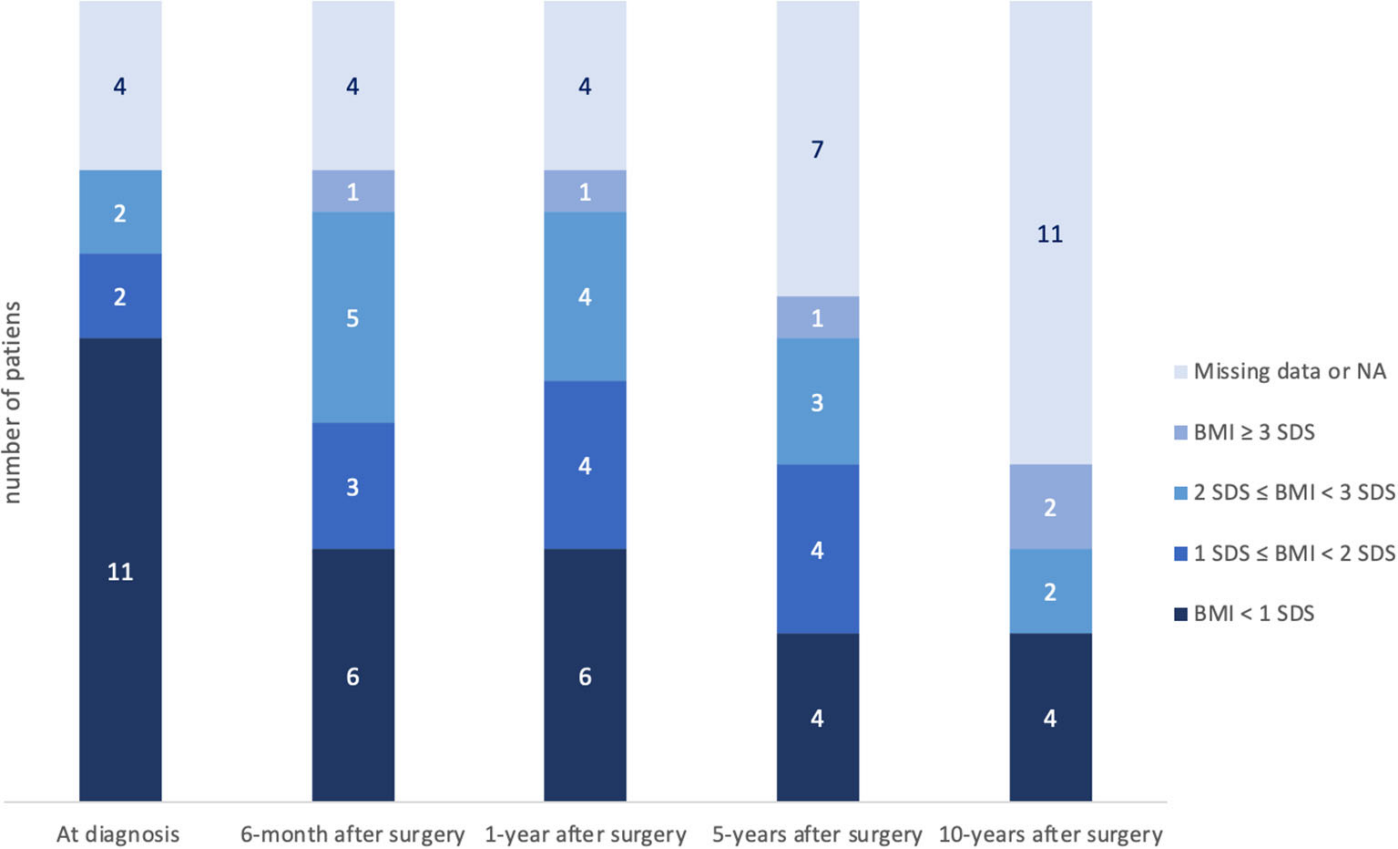
Evolution of BMI at diagnosis and after surgery. Obesity was defined with BMI ≥ 2 SDS. Thirteen percent of patients (2/15) were already obese at the time of diagnosis. There was no statistically significant difference between the preoperative BMI and the 6-month postoperative BMI (p=0.273). *BMI, body mass index; SDS, standard deviation score; NA, not applicable*.

The evolution of BMI at diagnosis and postoperatively is summarized in **Table 3**; **Figure 3**.

**Table 3:** Body mass index at diagnosis and after surgery.

BMI in SDS	Median [range]
At diagnosis	0.2 [- 2.8 and 3]
6-month after surgery	1.3 [- 2.2 and 4.2]
1-year after surgery	2 [-2 and 3.9]
5-years after surgery	1.5 [- 2.3 and 3.4]
10-years after surgery	1.5 [-0.7 and 3.4]

BMI expressed in SDS at diagnosis, 6 months, 1 year, 5 years, and 10 years after the first surgery.

BMI, body mass index; SEM, standard error of the mean.

At diagnosis, 2 of 15 patients (13%) had obesity, and 6 months after surgery 6 of 15 patients (40%) were affected by obesity. Of note, the number of patients with a BMI > 3SDS continued to increase during long-term follow-up. However, there was no statistically significant difference between the preoperative BMI and the 6-month postoperative BMI (p=0.273). There was no association between obesity (at 6 months and 1 year postoperative) and the type of tumor resection (total, subtotal) at first surgery (p=0.592 and p=0.286, respectively). There was no association between BMI at diagnosis and preoperative tumor volume (p=0.225).

### 3.6 Visual impairment

At diagnosis, 4 of 15 patients (27%) had papillary edema, and fifteen (79%) had visual impairment. At the last follow-up, 15 (79%) had visual impairment. When visual disturbances were found, the average preoperative volume was higher (p=0.039). Ten patients with visual disturbances confirmed by an ophthalmological examination preoperatively retained visual disturbances postoperatively at the last follow-up. Two patients without visual disturbances at diagnosis developed visual disturbances postoperatively. In one patient, visual disturbances at diagnosis disappeared after tumor resection. Among the fifteen patients with visual disturbances on preoperative ophthalmological examination, only nine (60%) had complaints at the time of diagnosis.

## 4 Discussion

The present study provides an extensive description of the management of pediatric patients diagnosed with CP during a 33-years period. To our knowledge, this study is the only available research involving Belgian pediatric population covering the endocrinological and visual outcomes.

The characteristics of the tumors of our patients, including solid and cystic components, the presence of calcifications, and their location, are consistent with the literature (1, 2).

Literature has reported a peak incidence of ACP between 5 and 15 years old (1, 5), which is consistent with our results.

In the literature, increased intracranial pressure manifestations are often the primary manifestations of the tumor (5, 16), followed by visual impairment (62-84%) and endocrine deficits (52-87%) (4, 16). Indeed, headache and vomiting were the predominant symptoms in 63% of our patients, followed by visual impairment (47%), growth failure (26%), symptoms of AVP-D (16%) and weight gain (10.5%).

The median delay between symptom onset and diagnosis was 6 months, again consistent with the literature (1, 4, 5). *Hoffmann et al.* (4) found that only hydrocephalus significantly shortened the diagnostic delay. This diagnostic delay (sometimes years) is attributed to the nonspecific and insidious symptoms, which, along with the slow growth of CP, lead to misdiagnosis (4).

Due to its anatomical relationships with the anterior pituitary and pituitary stalk, patients with CP may present with various hormonal deficiencies. Surgical resection of the CP usually worsens these hormonal deficits, as shown in our study. At diagnosis, 53% of our patients exhibited at least one hormonal deficiency (40 to 87% in the literature (2)) whereas 100% exhibited at least one hormonal deficiency 10 years after diagnosis. Our results are consistent with a few national studies involving childhood CP (17, 18).

Of note, 61% experienced growth failure (75% in (1)), at a median age of 5 years 5 months (range [1 year 2 months – 13 years 3 months]) which corresponds to a median time lag of 1 year 4 months (range [0 month – 5 years 6 months]) before diagnosis. This confirms that growth failure is one of the primary manifestations of CP, which frequently starts years before diagnosis but nonetheless goes unrecognized (19).

**Figure 4:**
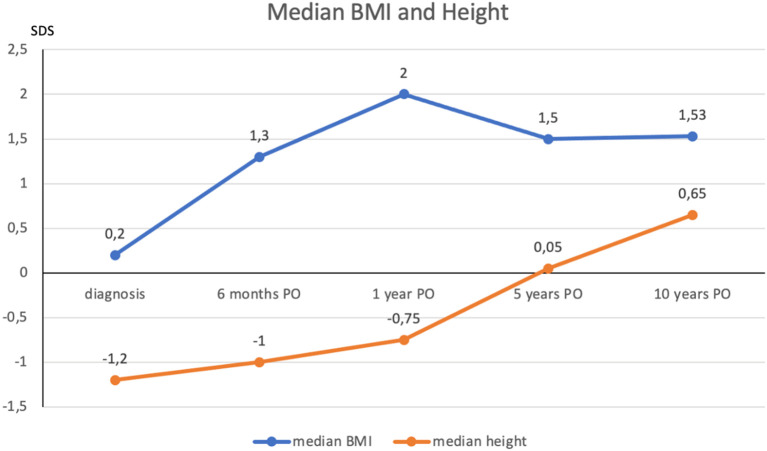
Evolution of median BMI and height at diagnosis and after surgery. BMI and height expressed in SDS. The postoperative occurrence of weight gain was increased at 6-months and 12-months after surgery, then reaching a plateau during the rest of the follow-up. Recombinant growth hormone replacement therapy is usually initiated between 6 months and 1 year after the completion of oncological therapy. *PO, postoperative; BMI, body mass index; SDS, standard deviation score*.

Hypothalamic dysfunction (1) due to tumor involvement or surgical damage to the hypothalamus plays a major role in long-term morbidity. Thirteen percent of our patients were already obese at the time of diagnosis (12 to 19% of patients in *Otte and Müller* (2), 30% in *Müller et al.* (1)), but only 10.5% complained of weight gain before diagnosis. As shown in **Figures 3**, **4**, body mass index increased at 6 months and 12 months after surgery and then plateaued during the rest of the follow-up.

Visual symptoms, including low visual acuity and visual field defects, are present in 70 to 80% of affected children (1, 16). Visual impairment was confirmed by an ophthalmological examination in 79% of our patients, but only 47% (9/19) had spontaneously reported visual impairment at diagnosis.

A thorough evaluation of visual function (visual field tests, visual acuity assessment, optical coherence tomography of the optic nerve and of the macular ganglion cell layer, fundoscopy) at diagnosis and during follow-up is necessary to assess the impact of the CP on vision and plan appropriate management (16, 20). Indeed, visual deficit may develop in the course of therapy, as shown in our study. A national Danish study (studying both childhood and adulthood CP) (20) shows that tumor recurrence is a strong predictor of decline in visual acuity.

Recently, a shift has occurred in neurosurgical management, prioritizing maximal safe resection over GTR to preserve critical structures and reduce complications (2, 5). *Tan et al.* (21) showed that a more conservative surgery reduced the prevalence of hormone deficiencies, including AVP-D, but this approach was not associated with a decrease in hypothalamic and visual morbidities, which remain significant.

Defining and measuring preoperative hypothalamic involvement is essential when planning the surgical strategy in pediatric patients. Müller’s classification is used to define this involvement although it makes no distinction between tumor invasion and destruction/compression of the structure. The grading system reported by Puget is another interesting way to classify the tumor according to the degree of hypothalamic involvement on preoperative MRI: grade 0 when there is no hypothalamic involvement, grade 1 when the tumor is adjacent to or displaced from the hypothalamus and grade 2 when the hypothalamus is no longer visible (22).

Our study showed that most patients experienced tumor recurrence or progression after the initial surgery, with a median time lag of 22 months. Subsequent treatment options included a second surgery, postoperative radiotherapy, and a combination of both. Proton therapy and gamma knife were used as radiotherapy modalities in the studied patients for 73% and 27% of our patients, respectively.

Radiotherapy is a highly effective approach for achieving long-term disease control in children (5). This has improved patient outcomes (23). There is no consensus on the optimal timing for radiation therapy after incomplete resection (immediately after surgery vs. at the time of its progression); thus, the decision is made on a case-by-case basis by a specialized multidisciplinary team (1, 21, 24). In our hospital, the practice today is to perform proton therapy (since 2011) or gamma knife therapy on patients with recurrence or progression of tumor volume greater than 25% after a multidisciplinary discussion. Proton therapy or gamma knife offers the benefit of delivering a significantly lower radiation dose to nontarget tissues than photon therapy, which could yield long-term clinical advantages for children with CP (15, 25, 26). It remains important to emphasize that cranial irradiation is associated with toxicities, including hypopituitarism. At long term, the risk of a second brain tumor remains controversial, mainly due to their rarity and the long latency period needed for them to manifest (27).

For patients with signs of elevated intracranial pressure or vision loss, urgent surgical decompression is recommended (1). In our cohort, relying on the expertise of the neurosurgical team, the preferred emergency neurosurgical management involved the placement of an external ventricular drainage and the debulking of the lesion to relieve the third ventricle.

As CPs are complex tumors, varying in size, composition (cystic and solid), and localization, the choice of the best clinical management (surgical indication, follow-up, surgical route) must take into account the surgeon’s experience and be individualized, considering the risks and benefits for the patient.

Treatment of childhood CPs varies across the world, and there is no consensus standard treatment (23). Intracystic therapies, such as interferon-α, are not used in our hospital but can be useful in the youngest patients to postpone radiotherapy, though they are limited to the cystic portion (1, 28). Moreover, radiotherapy agents such as yttrium-90 may be associated with irreversible neurotoxicity or even death and have not proven to be consistently efficacious (1). Three patients referred from Algeria with complex CP had grade 2 tumors at diagnosis. One received intracystic radioactive yttrium injection, 1 underwent intracystic interferon-α treatment, and 1 underwent GTR in Algeria. Importantly, these patients, who received prior care in their home country, had a worse outcome, especially visual, than the children initially treated in Belgium. Facilitating direct referrals of children with CP to specialized reference centers is crucial for optimizing their care and outcomes.

Hormone replacement therapy is administered on a personalized basis, according to each patient’s specific hormonal deficiencies. The doses are regularly adjusted based on the patient’s response and hormonal levels.

Recombinant growth hormone replacement therapy is crucial for children with CP to promote normal growth and body composition. *Miao et al.* (29) report that progression-free survival showed no significant difference between patients who received and did not receive rGH. European guidelines currently recommend growth hormone replacement therapy one year after surgery (30). In a recent retrospective study conducted by *Nguyen Quoc et al.* (31), no association was found between the timing of GH replacement therapy after childhood-onset CP treatment and the risk of recurrence or tumor progression, suggesting that GH replacement therapy can be initiated 6 months after the last treatment for CP.

Hypothalamic obesity is one of the main challenges in the management of patients with CP. It requires a comprehensive and integrated approach that encompasses dietary control, therapeutic education, and promotion of physical activity (32). In addition to a reduced basal metabolic rate, these patients often have a disrupted circadian rhythm and daytime somnolence. Various drugs have been proposed, including methylphenidate, oxytocin, metformin, and GLP1 analogs (33), though these pharmacological treatments are still debated (34).

The occurrence of AVP-D was clearly increased after surgery in our cohort, requiring desmopressin in all cases. Adapting the treatment with DDAVP can be complicated initially and requires patient education (35). Data regarding the neuropsychological follow-up of patients were not included in this study due to their limited availability. Recent studies have emphasized the importance of assessing the cognitive outcomes of patients with CP (36).

Study limitations. This retrospective study of pediatric patients with a rare tumor, spanning thirty years of care and follow-up, has obvious limitations, first due to missing data resulting from the long study period, loss of follow-up during the transition from pediatric to adult care and Algerian patients returning to their home country. Second, the limited number of cases in this series makes reduces the likelihood of highlighting any statistically significant effect.

## 5 Conclusion

CP is an extremely challenging brain tumor despite its benign histological nature. Endocrine, visual, and cognitive comorbidities lower the quality of life of patients affected by CP and their families. This benign tumor should be seen as a chronic disease requiring a lifelong multidisciplinary follow-up and hormonal substitution therapy. Hence, the goal of surgical resections not to achieve the maximum extent of surgical resection “at all costs”, but to preserve hypothalamic integrity whenever possible. This clinical study highlights the fundamental importance of carrying out a complete assessment (radiological, ophthalmological, endocrinological, but also neurocognitive) at the time of diagnosis and during follow-up so that patients can benefit from the best possible care. In addition, given this high disease burden, the clinical team must address the psychological distress that goes along with this chronic condition and will inevitably alter both the quality of life and the adherence to treatment of the patient.

## Data availability statement

The original contributions presented in the study are publicly available. This data can be found here: PRJNA1098038.

## Ethics statement

This study was approved by the Ethics Committee of HUB (P2023/155).

## Author contributions

CM: Writing – original draft. CB: Writing – review & editing, Supervision. MP: Writing – review & editing. SL: Writing – review & editing. NG: Writing – review & editing. OD: Writing – review & editing. CH: Writing – review & editing. EB: Writing – review & editing. IS: Writing – review & editing, Resources. CF: Writing – review & editing. FV: Writing – review & editing. LL: Writing – review & editing, Resources. MR: Supervision, Writing – review & editing.
